# Magnetomyographic assessment of pelvic floor muscles compared to ultrasound during pregnancy

**DOI:** 10.14814/phy2.70266

**Published:** 2025-03-09

**Authors:** Sallie Oliphant, Luis Mercado, Eric R. Siegel, Crystal Jones, Heather Moody, Diana Escalona‐Vargas, Hari Eswaran

**Affiliations:** ^1^ Department of Obstetrics and Gynecology University of Arkansas for Medical Sciences Little Rock Arkansas USA; ^2^ Department of Biostatistics University of Arkansas for Medical Sciences Little Rock Arkansas USA

**Keywords:** Kegel, levator ani muscle, magnetomyography, power spectral density, pregnancy

## Abstract

Maternal birth injury contributes to future pelvic floor disorders, yet we possess an incomplete understanding of the levator ani muscles during pregnancy. We applied a noninvasive magnetomyography technique to characterize levator ani muscle activity in pregnancy with ultrasound and clinical exam. Magnetomyographic measures of levator ani muscle activity were collected using a noninvasive biomagnetic sensor from 53 pregnant women during rest and voluntary muscle contractions of varying intensity. Power spectral density was calculated using Welch's method to obtain the mean power of each Kegel exercise. Levator hiatus circumference was measured using ultrasound, and contraction strength was measured via the Brink scale. Magnetomyography data revealed a mean root mean square (RMS) rest of 39.7 ± 8.6 femtoTesla (fT) and Kegel of 52.9 ± 17.1 fT. Mean power spectral density (PSD) in log_10_ (fT^2^/Hz) was 0.9 ± 0.2 at rest and 1.1 ± 0.2 during Kegel. Ultrasound measures of levator hiatus circumference were 13.3 ± 1.6 cm at rest and 11.6 ± 1.7 cm during maximum Kegel. Magnetomyographic correlations with levator hiatus circumference were stronger for amplitude and PSD at rest (−0.35 and −0.33) than for Kegel (−0.20 and −0.19). Magnetomyography‐based amplitudes of pelvic floor activity directly correlate with ultrasound levator hiatus circumference during rest and Kegel.

## 1 INTRODUCTION

The female pelvic floor muscles are critical to the maintenance of continence and for the support of the pelvic organs (Heilbrun et al., 2010). Previous work has demonstrated a strong link between maternal birth injury and the later development of pelvic floor disorders (PFDs) such as urinary incontinence, bowel incontinence, and pelvic‐organ prolapse (Memon & Handa, 2013). As many as one in four women will suffer from a PFD in her lifetime and as many as one in five will undergo corrective surgery (Wu, Matthews, et al., 2014; Wu, Vaughan, et al., 2014). These conditions often present years and even decades after pregnancy; however, vaginal deliveries, especially those with operative extraction, are thought to be the inciting event in the causal pathway for many women (Blomquist et al., 2018). These disorders are often costly, difficult to treat, and highly impactful to quality of life. Thus, efforts to identify and ideally modify contributing factors have been underway, though with limited success (Baud et al., 2020; Bazi et al., 2016).

The levator ani muscles (LAMs) of the pelvic floor, along with the perineum and anorectum, are vulnerable to injury during vaginal birth, and birth injury has been correlated with long‐term PFD risk. In addition, more significant injury to the perineum and anorectum (obstetric anal sphincter injury) has been correlated with long‐term PFD risk (Baud et al., 2020; Memon & Handa, 2012). The LAMs are unique among human skeletal muscles, as they must maintain resting tone to support the pelvic organs, contract during increases in abdominal pressure to maintain continence, relax for urination and defecation, and stretch significantly during vaginal birth (Ashton‐miller & DeLancey, 2007). Though active skeletal muscle has been shown to undergo injury at the sarcomere level with as little as 50% stretch, the pelvic floor must stretch as much as 150%–300% during term vaginal birth in order to accommodate the passage of the fetal head (Brooks et al., 1995; Lien et al., 2004; Svabík et al., 2009). This stretch is thought to incite near universal microinjury to the LAMs, with many women sustaining macroinjury including full and partial thickness tears and muscular avulsions (Pipitone et al., 2021). During pregnancy, the female body undergoes remarkable physiologic changes, including significant adaptations to the pelvic floor to allow for vaginal birth. Relaxin production, along with other hormonal factors, increases muscular and connective tissue laxity by altering the ratios of elastin and collagen, which may allow the levator muscles to undergo significant stretch with minimal long‐term damage (Dehghan et al., 2014).

Despite the established importance of the LAMs in the maintenance of pelvic floor health, we have a limited understanding of the changes these muscles undergo during pregnancy and postpartum. Previous work has utilized electromyography (EMG), magnetic resonance imaging (MRI), ultrasound, and vaginal pressure measures to explore the LAMs (Bø et al., 2022; Deng et al., 2023; Pipitone et al., 2021; Wu et al., 2023). Our team has developed a novel protocol to capture the activity of the LAMs in the resting and contracted states using magnetomyography (MMG), a passive measure of the magnetic fields created by muscle fiber depolarization, which provides a spatiotemporal map of muscular activity (Escalona‐Vargas et al., 2019, 2021). The ability of MMG to collect signal data during the passive state is particularly useful in studying the LAMs, as LAM resting tone is critical to normal function and support of the pelvic viscera (Worman, Stafford, Cowley, & Hodges, 2023). The objective of this study was to correlate passive and active LAM activity in third trimester pregnancy via MMG with the more traditional measurement techniques of ultrasound and clinical exam. Although we have shown that MMG is similar to surface EMG measures, this is the first attempt to correlate a functional measure of pelvic floor activity against an imaging modality.

## 2 STUDY DESIGN

Following institutional review board approval (IRB# 202706), we recruited a cohort of gravidas receiving routine prenatal care with our tertiary care hospital from October 2021 to March 2024. Women eligible for recruitment included adults with an ultrasound‐confirmed singleton gestation, and all enrolled women underwent a third trimester study visit. Participants were excluded for a history of prior vaginal birth or cesarean birth after the onset of second‐stage labor, any known lethal fetal anomaly or maternal/fetal contraindication to trial of labor, or planned delivery prior to term. Sixty‐six women who met the eligibility criteria provided written consent to participate and received a small monetary compensation for participation.

Demographic and clinical data was obtained via medical record chart extraction. Study visits consisted of measurements using translabial 3D/4D ultrasound imaging of the levator hiatus, EMG recordings capturing pelvic floor and accessory muscle groups, Brink assessment of Kegel strength, and MMG recordings at rest and with Kegel activity. Participants were asked to perform voluntary LAM contractions, known as Kegel exercises. While MMG and EMG recordings were done simultaneously, ultrasound and Brink measurements were collected separately during the same study visit.

### 2.1 Ultrasound

**FIGURE 1:**
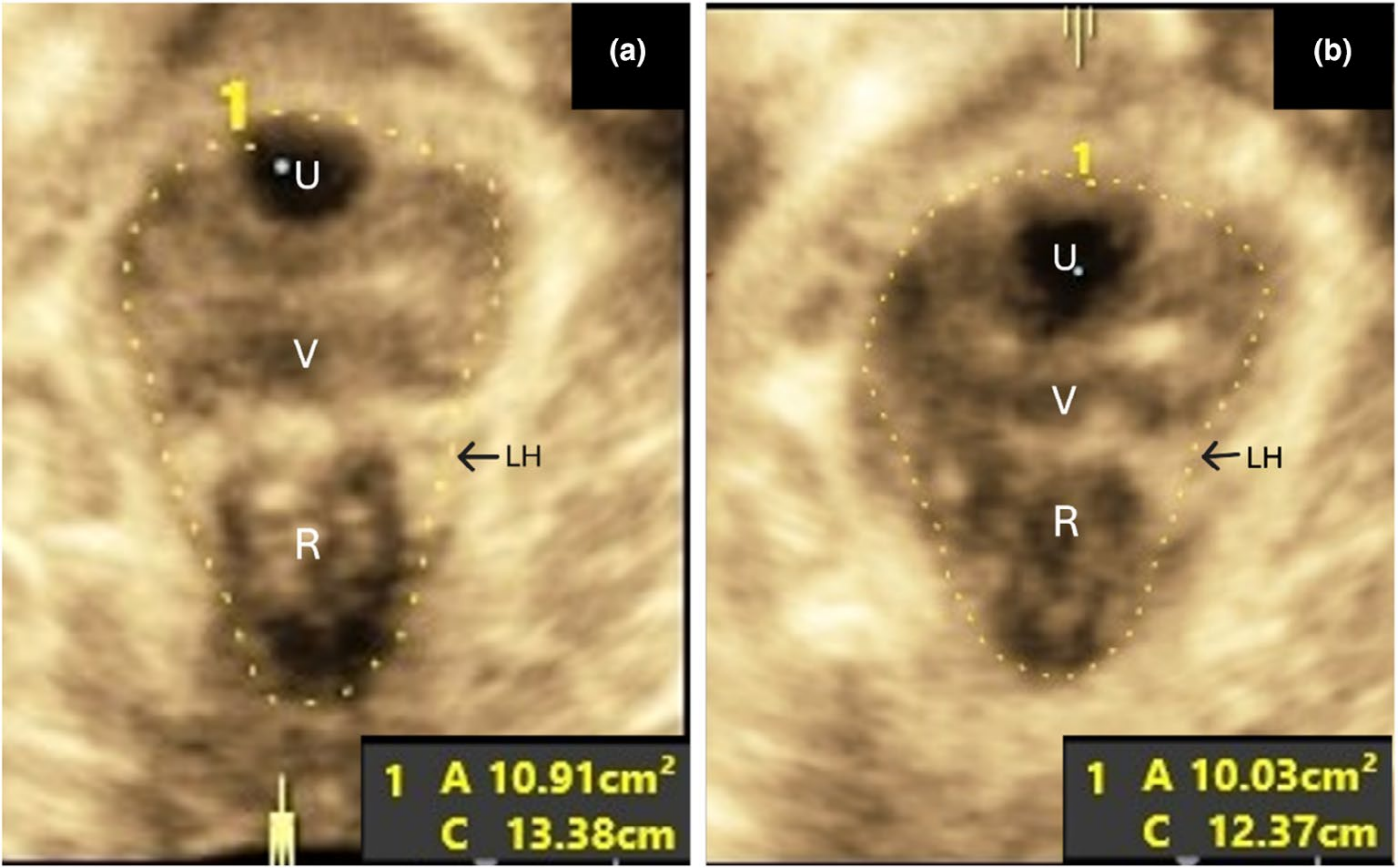
Axial plane 3‐D translabial/transperineal ultrasound image of LAM rest and contraction (Kegel) showing representative circumference and area measurements in the plane of minimal dimensions.

Ultrasound assessment of the pelvic floor was performed by a trained medical professional using a 3.5‐6 MHz curved array transducer. The transducer was covered using a gel‐filled condom, and images were obtained by translabial/transperineal ultrasound. A 3/4‐dimensional cine loop of the midsagittal view of the pelvic floor from the resting state through maximal contraction (Kegel) was obtained with the patient placed in a dorsal modified lithotomy position. Images were configured to display the plane of minimal dimensions for measurement (Dietz & Shek, 2009). Levator hiatus circumference and area were independently measured by two trained examiners with high inter‐rater reliability, and the average of these measurements was utilized for analysis. The representative measurement of the levator hiatus circumference and area is shown in Figure 1a,b for the axial plane 3‐D ultrasound image of LAM rest and contraction, respectively. The levator hiatus is the opening created by the funnel‐shaped muscles that form the pelvic floor. The hiatus is defined by the inferior pubic rami anteriorly and the medial component of the levator ani muscle laterally and posteriorly.

### 2.2 Brink scoring

Brink score is currently a standard evaluation of pelvic floor strength, measured during a clinical exam on an ordinal scale. This measure grades pelvic floor muscle contraction strength, symmetry, and sustainability using digital vaginal exam and the ability of the subject to isolate and voluntarily contract the LAM (Brink et al., 1989). In this study, the pelvic floor muscle strength was assessed digitally via vaginal exam using the Brink scoring method by two trained medical professionals. Brink score B1 (squeeze pressure 0–4) was analyzed for the group and used for comparisons.

### 2.3 Magnetomyographic and Electromyographic signals

**FIGURE 2:**
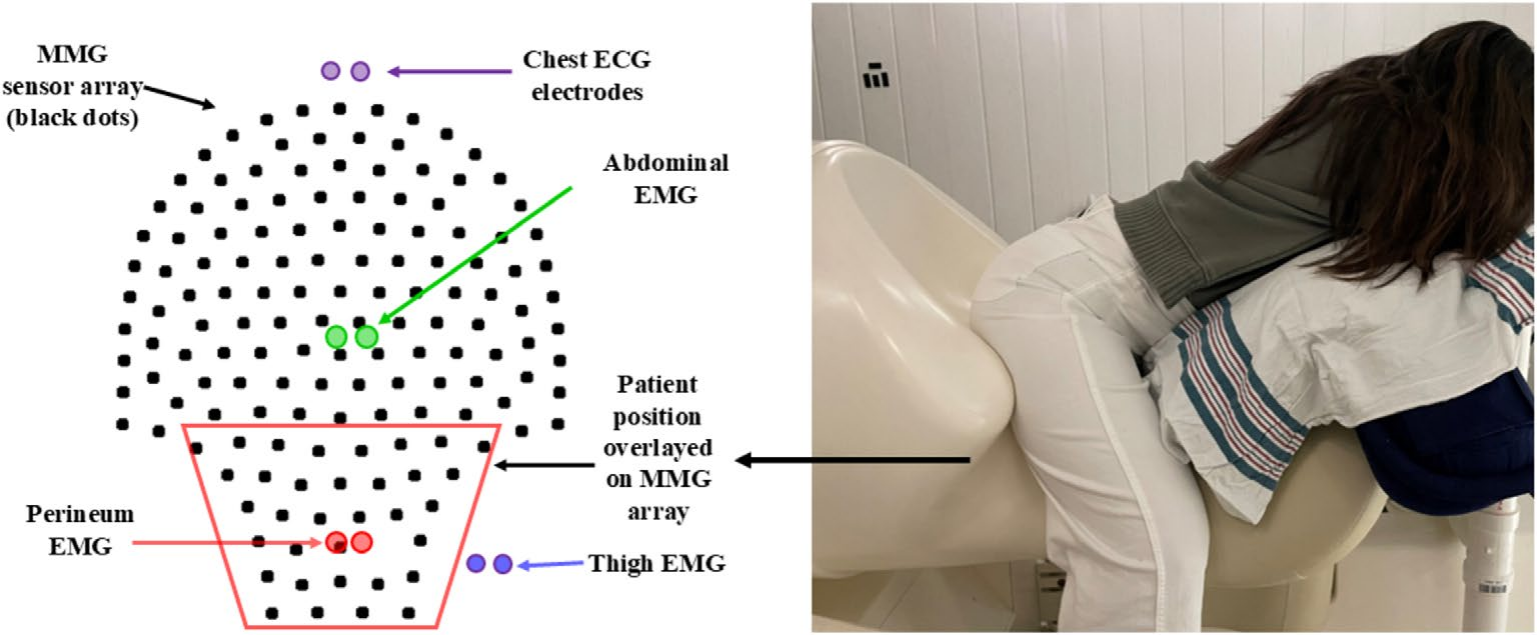
The patient is positioned in the SARA system for recording the MMG signals from pelvic floor muscles. The lower half of the sensor array corresponds with pelvic floor musculature. The participant provided written consent for the use of this image.

Magnetomyography is a technique that is capable of noninvasively recording signals from muscle activity from both smooth and skeletal muscles (Escalona‐Vargas et al., 2021). This method is based on the principle that any electrical source that can be recorded with EMG has a magnetic homologue, MMG, which is measured by biomagnetic sensors called SQUIDs (Superconducting Quantum Interference Device). Using this technique, the pelvic floor muscle signals were assessed at each visit using the UAMS SQUID Array for Reproductive Assessment (SARA). The SARA device quantifies pelvic floor muscle activity by isolating and recording the MMG signals generated by pelvic floor muscle‐fiber depolarization. Paired surface‐patch EMG electrodes were placed on the ventral abdominal wall (rectus abdominus), upper lateral thighs (gluteal muscles), and perineum (LAM). The EMG was purely used for visualization purposes of the abdominal, perineum, and thigh muscles during the exercise sequence. Participants were positioned on the sensor array in a manner designed to interface directly with the pelvic region (Figure 2). Twenty‐one MMG sensors from the lower half of the array were selected for analysis to correspond to the anterior, posterior, and lateral borders of the muscle group of interest.

Women performed a pre‐established sequence of voluntary muscular activities including gradated Kegels with subjectively small (KegSmall), medium (KegMed), and large (KegLarge) intensities for 10 s, with an intervening 10 s rest between each exercise. The sequence continued with 10 s of isolated abdominal and thigh contraction. MMG data was collected at a sampling rate of 1.25 kHz, band‐pass filtered between 20 and 200 Hz using a 4th‐order Butterworth filter, and notch‐filtered at 60 Hz for ambient noise removal. SARA sensors were selected as shown in Figure 2 (left) for analysis given positional proximity to LAMs. The open‐source Brainstorm software (Tadel et al., 2011) was used to discard noisy channels by visual inspection of all preprocessed data to ensure an adequate signal. We applied an advanced signal processing method referred to as frequency dependent subtraction (SUBTR) (Vrba et al., 2012) for attenuation of thigh activities.

To improve the signal‐to‐noise ratio (SNR), Independent Components Analysis (ICA) was used on the selected MMG channels. Only 10 independent components were calculated using ICA, and those components that did not include a visible muscular burst were removed from the signal. Ten‐second MMG epochs corresponding to each Kegel exercise were extracted with baseline correction using the previous 10 s rest period. The 4‐s window in the middle of the epoch (midpoint of the Kegel activity) was used for analysis. The mean MMG amplitude between channels was selected to describe the gross innervation input of the LAM muscle. The MMG amplitude is related to the strength of the muscle contraction, and lower pelvic MMG amplitude—lower is the force generated by the muscle. Power spectral density (PSD) was calculated using Welch's method to obtain the mean power of the Kegel exercise for each participant. PSD provides the power contained in each frequency within the muscle activity, which is related to the physiological status of the muscle. In general, PSD shifts to lower frequency as the muscle fatigue increases, thus providing a quantitative measure of pelvic floor muscles during rest and exercise.

### 2.4 Statistics

Maternal and gestational age were summarized as mean ± SDs (standard deviations), whereas race was summarized as percentages: Hispanic, Non‐Hispanic Black, and Non‐Hispanic White. Circumferences of the levator hiatus are expressed in centimeters (cm). MMG amplitudes are expressed in femtoTeslas (fT). The MMG PSDs are expressed in squared fT per Hertz (fT^2^/Hz). The levator hiatus circumferences, MMG amplitudes, and MMG PSDs were summarized by exercise state (at rest versus during a Kegel) as mean ± SDs. The paired differences in subjects' levator hiatus circumferences during a Kegel versus at rest were expressed as mean ± SDs. The paired ratios of MMG amplitudes and PSDs during a Kegel versus at rest were transformed to SNR in decibels (db) following established methods; the resulting paired differences were summarized as mean ± SDs. The associations of MMG amplitudes and MMG PSDs with levator hiatus circumferences, both at rest and during a Kegel, were visualized as scatter plots and quantified as Pearson correlation coefficients. Based on Cohen (1992), correlation coefficients are descriptively interpreted as small, medium, or large, respectively, if their magnitudes are in the neighborhood of 0.1, 0.3, and 0.5. Statistical null‐hypothesis tests were not performed, and *p* values are thus not reported.

## 3 RESULTS

Of the 66 women who were consented, seven were excluded due to incomplete data collection and six were excluded due to low signal‐to‐noise ratio, leaving 53 subjects to include in further data analysis. Subjects' mean age was 24.3 ± 4.9 years, with 75% self‐identifying as White, 13% as Black, and 21% as Hispanic. The mean gestational age at the time of the study visit was 30.2 ± 1.6. From those 53 subjects, their gravidity and parity consisted of: 45 gravida(G1), 4 G2 parity(P0), 1 G3 P0, and 3 G2 P1.

**FIGURE 3:**
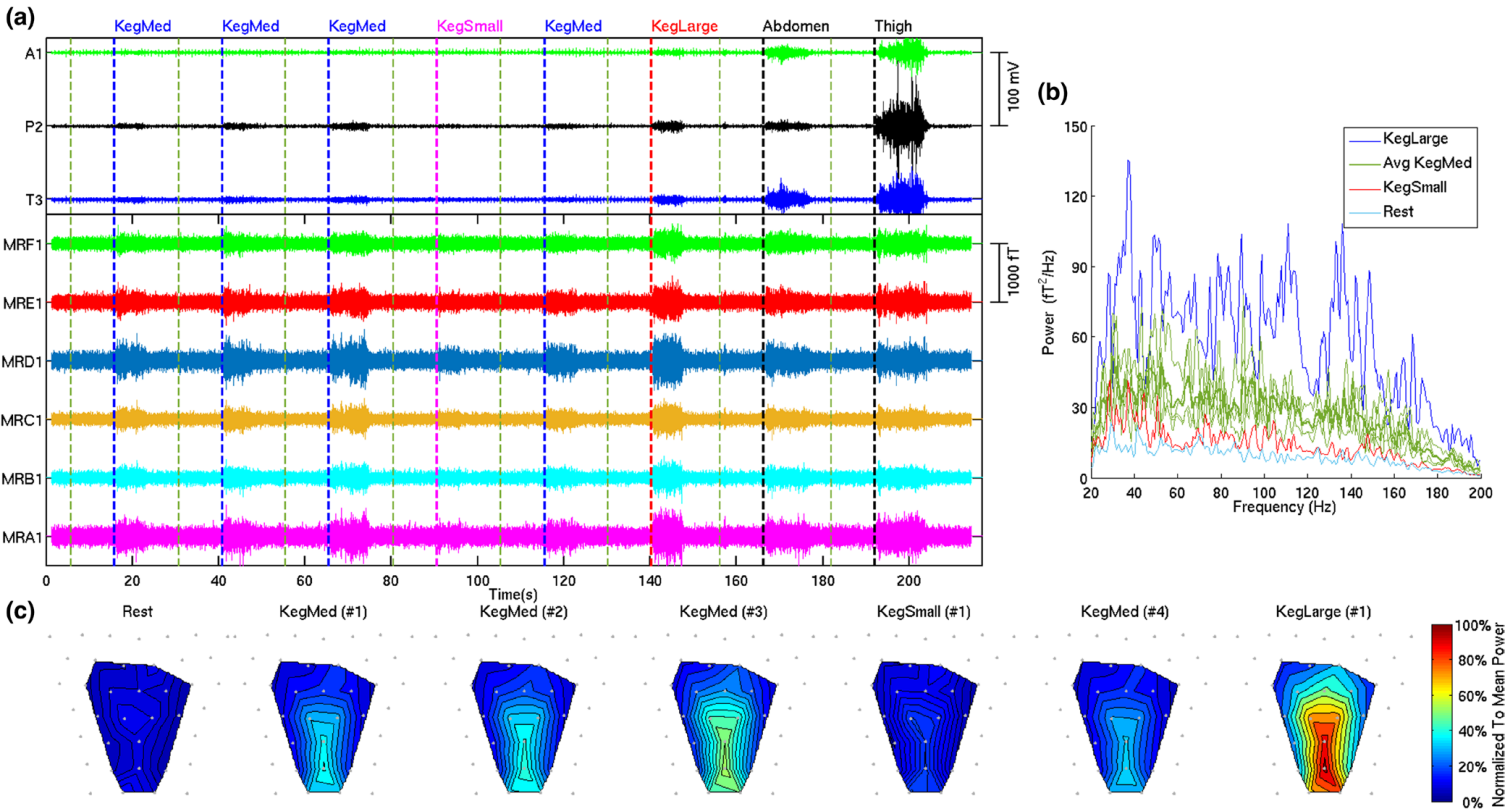
(a) The six representative MMG channels are labeled as MRA1, MRB1, MRC1, MRD1, MRE1, and MRF1 cover the pelvic musculature. And EMG channels are labeled as A1 (abdomen), P2 (perinium), and T3 (thigh). The exercise sequence performed by the participants are marked on the figure. (b) The PSD averaged across all MMG channels for each type of Kegel exercise is expressed in fT^2^/Hz. (c) Power maps representing means of power across frequencies of MMG activities by sensor location. The color map depicts the strength of the MMG for each of the exercise routine overlayed on the sensor array covering six rows of MMG channels. The color gradation ranges from blue to red representing lowest to highest normalized mean power respectively.

Figure 3a shows a representative recording of six MMG channels (labeled with prefix M) and three EMG channels (A1‐Abdomen, P2‐Perineum, and T3‐ Thigh electrode pairs) capturing the exercise sequence. These six MMG channels are from the lower half of the sensor array (Figure 2 – left) which corresponds to pelvic floor musculature, with one channel from each row shown for representative purposes. Also, the figure shows correspondent signals from an EMG electrode pair on the perineum (P2), capturing surface‐level LAM activity simultaneously. The additional 2 EMG channels reflect accessory muscle activity of the abdomen (A1) and thigh (T3). The EMG data provide visualization of the localized activity during the exercises. Figure 3b shows the PSD in units of fT^2^/Hz averaged across all the 21 MMG channels for each type of Kegel exercise and rest. Figure 3c shows the color‐coded power maps representing means of power across frequencies of MMG activities overlaid by the sensor locations.

**FIGURE 4:**
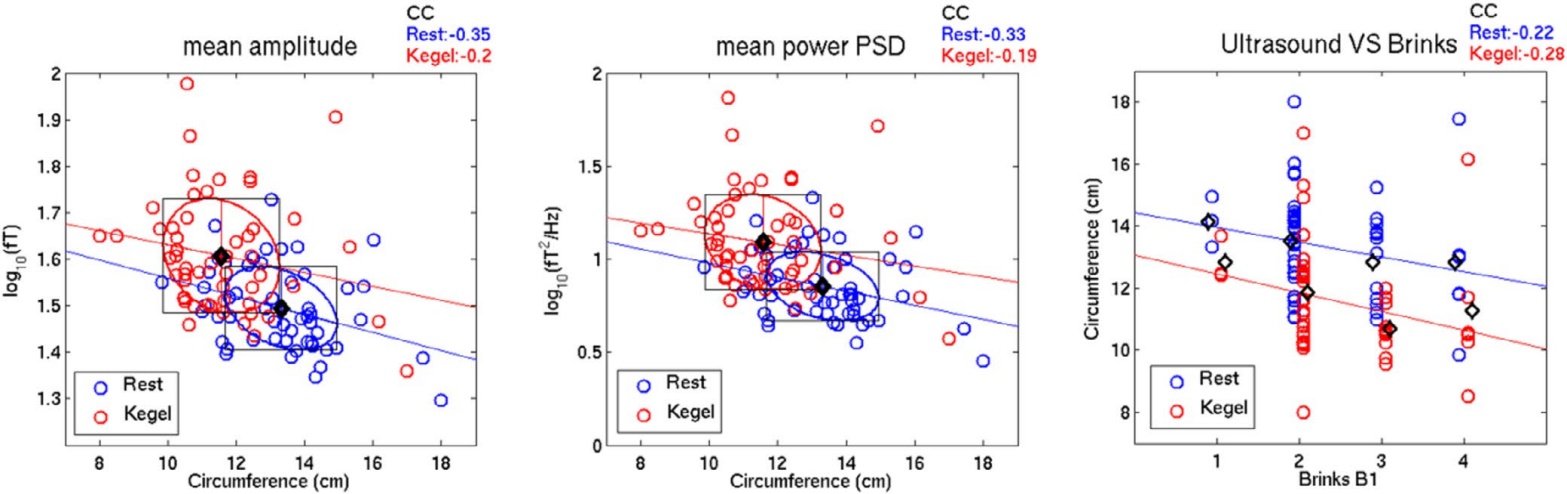
Correlation plots of mean amplitude (left panel), mean power PSD (middle panel), and Brink score (right panel) versus ultrasound levator hiatus circumference (all 3 panels) during rest (blue colors) and during Kegels (red colors). Colored open circles denote individual data points, while black open diamonds denote group means. The colored ellipses with circumscribed black rectangles around the group means respectively denote each group's bivariate 1‐SD ellipse with univariate ±1‐SD distances in the vertical and horizontal directions. The colored lines through the group means are group‐specific regression lines whose standardized slopes (i.e., correlation coefficients or CCs) are provided above the top right corner of each panel.

Overall, magnetomyographic data for the cohort revealed: Mean RMS 39.7 ± 8.6 (rest) and 52.9 ± 17.1 (Kegel); mean amplitude 1.5 ± 0.1 log_10_ (fT) (rest) and 1.6 ± 0.1 log_10_(fT) during Kegel. PSD had means of 0.9 ± 0.2 log_10_(fT^2^/Hz) at rest and 1.1 ± 0.2 log_10_(fT^2^/Hz) during Kegel. Ultrasound measures of levator hiatus circumference were 13.3 ± 1.6 cm at rest and 11.6 ± 1.7 cm during maximum Kegel. Figure 4 depicts correlation plots of mean amplitude and PSD during rest and Kegel versus ultrasound levator hiatus circumference. Correlations with circumference were stronger at rest for amplitude and PSD (−0.35 and −0.33, respectively) than for Kegel (−0.20 and −0.19, respectively). Median B1 pressure score was 2 (IQR 2,3). The correlation values, shown in Figure 4, between the circumferences and the Brink scores were also small in magnitude (−0.22 for rest and −0.28 for Kegel).

**FIGURE 5:**
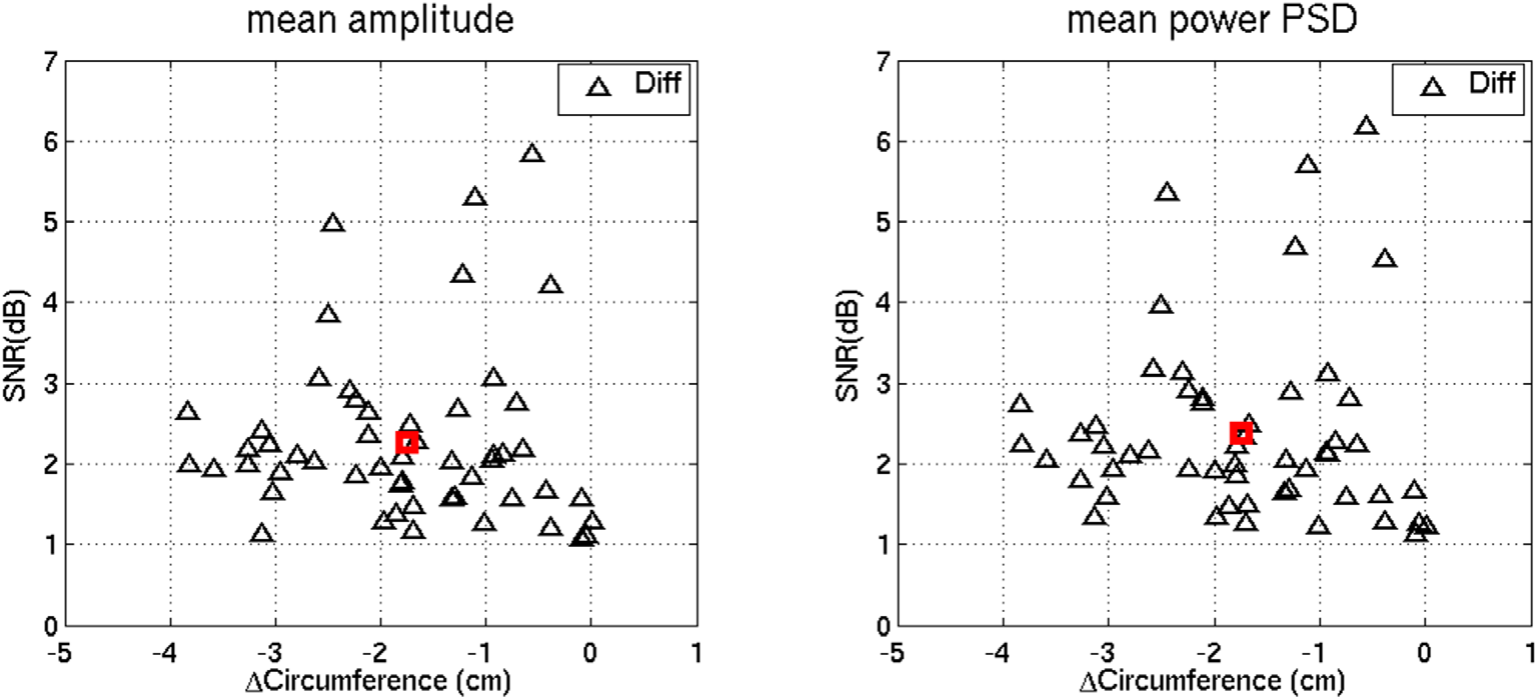
Scatter plots of the changes (SNR in dB) in the mean amplitude (left panel) and mean power PSD (right panel) between rest and Kegel versus the corresponding changes in ultrasound levator hiatus circumference (“ΔCircumference” in cm, both panels). Black triangles show the changes (“Diff”) of individual patients, while red squares show the mean changes.

The mean amplitude change between Kegel and rest was 2.3 ± 0.9 db and the mean PSD change was 2.4 ± 1.0 db. The mean change in circumference between rest and Kegel was a decrease of 1.7 ± 1.0 cm. Figure 5 depicts the scatter plots of the decibel increase in power versus the reduction in circumferences.

## 4 DISCUSSION

The body's control of pelvic floor muscle tone is integral to regulating a number of pelvic functions such as continence and childbirth (Worman, Stafford, Cowley, Prudencio, & Hodges, 2023). This regulation allows the pelvic floor muscle complex to maintain the integrity of the pelvic region and provide support for pelvic organs by producing myoelectric activity even at rest (Worman, Stafford, Cowley, Prudencio, & Hodges, 2023). The most notable finding of this study is the moderate correlation between the MMG mean amplitude and mean power PSD of resting activity of the pelvic floor musculature with ultrasound circumference measurements. Surprisingly, however, Brink scoring did not show a strong relationship with ultrasound hiatal circumference measurements, possibly due to the Brink scale not reflecting real clinical differences in pelvic floor muscular strength (FitzGerald et al., 2007). On the other hand, the Brink score retained a positive correlation with the mean amplitude and mean PSD during rest and Kegel, similar to our previously reported study (Mercado et al., 2024). Conventional measures of pelvic floor health focus on the active phase of muscular contraction, the strength of the Kegel exercise. Though measures such as baseline genital hiatus, Brink scoring, and resting EMG activity can provide some insight into the pelvic floor, MMG offers a unique spatiotemporal view of the resting activity of the LAMs. The MMG signals display various levels of resting and voluntary muscle contractions in units of amplitude. These changes can then be used to describe muscle activity and compute the mean power spectrum as a function of frequency.

We reduced crosstalk by using MMG sensors closer to the possible recruited muscles of the thigh during voluntary contractions. These processed signals were then used to compile a baseline quantification of pelvic floor resting and active muscle tone by analyzing the magnitudes at various intervals of rest and contraction. A larger correlation between MMG and ultrasound was seen with rest than with Kegel measurements. Variation was seen in resting amplitudes between individual participants that correlated with hiatal circumference measurements with ultrasound. By using spectral analysis, we were able to measure the strength of the signal as a function of frequency, thereby allowing for the assessment of the magnitude of energy contained in muscle activity at various stages of rest and Kegel. Our data did show that MMG amplitudes of LAM activity directly correlate with ultrasound circumference measurements of the hiatus during rest and Kegel. This further suggests that MMG allows for reliable quantitative functional assessment of LAM activity.

Previous reports have demonstrated significant changes in pelvic floor support and muscular anatomy during pregnancy (Sze et al., 2002). These variations in individual resting pelvic floor muscle tone may influence the LAM adaptation during childbirth and could potentially offer some insight into complication rates associated with pelvic floor birth injuries. Therefore, these findings may suggest that women with greater pelvic floor activity at rest during pregnancy could potentially be able to better maintain pelvic floor strength compared to those women with less activity at rest intrapartum, reducing the likelihood of experiencing postpartum symptomatic pelvic floor disorders. However, further investigation into this topic is needed.

A strength of this study is the inclusion of a variety of modalities to assess the pelvic floor, with the novel inclusion of MMG allowing unique insight into the resting activity of the LAMs. Due to the limited timeframe from which the data was collected, we were only able to achieve a functional assessment of the correlation of 3D transperineal ultrasound circumference measurements of the pelvic levator hiatus at rest and during voluntary contraction with MMG pelvic floor rest and active amplitudes during the third trimester of pregnancy. Several limitations are noted, including the relatively small sample size, missing data points, and the fact that subjects were not prescreened for the ability to correctly perform a Kegel maneuver. Furthermore, this study is another step towards showing the potential clinical utility of MMG. While the third trimester measures have yet to be incompletely defined clinical implications, we believe our future analysis comparing third trimester and postpartum measures will provide insight into injury and injury recovery as well as associated PFD symptoms. We hypothesize that MMG has the potential to allow for reliable assessment of factors related to birth injury and aid in the detection and evaluation of birth injury and recovery.

In the next phase of our study, we aim to compare measures of MMG signals from maternal pelvic floor muscles at rest and during active muscular contraction to compare within subjects for differences in MMG signal strength between pregnancy and postpartum. This data will then be correlated with birth outcome measures and maternal pelvic floor symptoms to detect injury by comparing women to their own predelivery baseline. The MMG injury detection data will then be compared against established pelvic floor measures with the aim of demonstrating a correlation between resting muscle tone and predisposition to pelvic floor injury.

## 5 CONCLUSION

Our descriptive data demonstrate that MMG can reliably assess the functionality of pelvic floor muscle activity in pregnancy. The moderate correlation of MMG resting pelvic floor muscle amplitudes with ultrasound hiatal circumference measurements points to the fact that MMG has the potential to allow for reliable assessment of factors related to birth injury and may aid in the detection and evaluation of birth injury and recovery. The level of tone associated with the resting state of the LAM may be a predictor of sustaining pelvic floor injury during childbirth. Therefore, women with greater pelvic floor activity at rest during pregnancy may be less likely to experience postpartum symptomatic pelvic floor disorders, experience less widening of the genital hiatus, and better maintain pelvic floor strength compared to those women with less activity at rest in the third trimester. Resting tone may also provide insight into complete versus incomplete muscle recovery following injury. However, further investigation into the correlations of resting LAM muscle tone with the incidence of pelvic floor birth injury, injury recovery, and associated clinical PFD manifestations is needed.

## FUNDING INFORMATION

This work is supported by US National Institutes for Health (NIH) grants 1R01EB031589 and 1R21HD091717.

## CONFLICT OF INTEREST STATEMENT

No conflict of interest to disclose other than the grant funding listed.

## ETHICS STATEMENT

The study was approved by the University of Arkansas for Medical Sciences Institutional Review Board (UAMS #202706). The research was conducted in accordance with the principles embodied in the Declaration of Helsinki and in accordance with local statutory requirements. All the participants signed an informed consent to participate in the study.

## PATIENT CONSENT STATEMENT

This study was approved by the Institutional Review Board, and all subjects provided written informed consent.

## Data Availability

The deidentified data will be available on request in accordance with institutional procedures and regulations.
